# Case Report: Refractory cold agglutinin disease with hypersplenism: efficacy of splenectomy in a patient treated with sutimlimab

**DOI:** 10.3389/fimmu.2026.1770676

**Published:** 2026-03-30

**Authors:** Antonella Sau, Daniela Onofrillo, Anna Caterina Russo, Gianfranco Di Prinzio, Anna Maria Quaglietta, Mauro Di Ianni, Bruno Fattizzo

**Affiliations:** 1 Department of Hematology and Oncology, Santo Spirito Hospital, Pescara, Italy; 2 Center of Transfusion Medicine, Santo Spirito Hospital, Pescara, Italy; 3 Department of Medicine and Sciences of Aging, G. D’Annunzio University of Chieti and Pescara, Chieti, Italy; 4 Hematology, Fondazione Istituto di Ricovero e Cura a Carattere Scientifico (IRCCS) Ca’ Granda Ospedale Maggiore Policlinico, Milan, Italy; 5 Department of Oncology and Haemato-oncology, University of Milan, Milan, Italy

**Keywords:** autoimmune hemolytic anemia, cold agglutinin disease, complement inhibitors, hypersplenism, splenectomy, sutimlimab, treatment resistance

## Abstract

Cold agglutinin disease (CAD) is a rare autoimmune hemolytic anemia characterized by complement-mediated extravascular hemolysis. The introduction of complement inhibitors, particularly sutimlimab, has significantly improved disease control. However, a subset of patients remains refractory to treatment, particularly those with inadequate bone marrow compensatory response to hemolysis, as well as those rare cases with hypersplenism. We report the case of a woman with refractory CAD who received multiple lines of therapy, including rituximab, bendamustine, sutimlimab, and pegcetacoplan, with suboptimal or transient responses. The clinical course was complicated by progressive splenomegaly and worsening cytopenias consistent with hypersplenism. Splenectomy was eventually performed, followed by re-initiation of sutimlimab. This combined intervention led to marked hematologic improvement, with resolution of anemia and thrombocytopenia, and sustained clinical response. While current guidelines do not recommend splenectomy in CAD due to the hepatic predominance of hemolysis, this case underscores the potential role of the spleen as a modifier of treatment resistance in selected cases. We discuss emerging literature on hypersplenism-associated refractoriness and emphasizes the importance of individualized treatment strategies in the management of complex or refractory CAD phenotypes. Given the rarity of CAD complicated by hypersplenism, large prospective trials may be difficult to conduct. Collaborative multicenter registries and prospective observational studies may represent more feasible approaches to define clinical and molecular predictors of response and to guide personalized therapeutic strategies in challenging subsets.

## 1 Introduction

Cold agglutinin disease (CAD) is a rare form of autoimmune hemolytic anemia (AIHA), accounting for approximately 15% of AIHA cases (1). It is characterized by monoclonal IgM autoantibodies—typically directed against the I antigen on red blood cells (RBCs)—which bind at low temperatures, leading to agglutination and classical complement pathway activation. The predominant site of hemolysis is the liver, where C3b-opsonized RBCs are cleared via Kupffer cells (1, 2).

Clinically, CAD presents with chronic anemia, cold-induced circulatory symptoms (e.g., acrocyanosis, Raynaud’s phenomenon), and episodic hemoglobinuria. Secondary CAD may arise in the context of infections, autoimmune disorders, or malignancy (3), whereas primary CAD is associated with bone marrow clonal B-cell disorders, often without overt lymphoproliferative disease (4). In both settings, complement activation is central to pathogenesis.

Treatment with corticosteroids is typically ineffective, and rituximab-based immunotherapy offers only partial or transient responses in many patients (1). The introduction of sutimlimab, a monoclonal antibody against C1s, has transformed the treatment landscape by selectively blocking the classical complement pathway, thereby reducing hemolysis and improving hemoglobin levels and fatigue (2, 5). Data from the CARDINAL and CADENZA trials confirmed its efficacy and safety, leading to regulatory approval and widespread clinical use (Reid et al., 2024). However, a subset of patients remains refractory, and real-world studies suggest heterogeneity in long-term response and tolerability (6).

In these complex cases, contributing factors include ineffective bone marrow compensatory response, which might be boosted by adding recombinant erythropoietin. Comorbidities may also contribute to reduced efficacy of complement inhibitors, particularly inflammatory ones which may increase the activation of the complement cascade. Splenic sequestration or hypersplenism represent rarer entities, mainly observed in patients with overt lymphoproliferation or in those with liver comorbidities. However, since splenectomy is not indicated in CAD, its use to restore or enhance responsiveness to complement inhibitors has never been explored.

Here, we report the case of a patient with severe, treatment-refractory CAD associated with hypersplenism, who experienced sustained hematologic recovery following splenectomy and continuation of sutimlimab. To our knowledge, this is the first reported case suggesting a synergistic effect between splenectomy and complement inhibition, supporting re-evaluation of splenectomy in selected CAD patients with confirmed features of hypersplenism.

## 2 Case presentation

A 56-year-old woman was diagnosed in 2018 with CAD following evaluation for chronic anemia and cold-induced acrocyanosis. The diagnosis was confirmed by multiple tertiary care centers with expertise in autoimmune cytopenias, based on a consistent profile of complement-mediated hemolysis, cold-reactive antibodies, and exclusion of clonal or autoimmune comorbidities. Laboratory workup revealed a mild IgG monoclonal gammopathy, a positive direct antiglobulin test (C3d-positive, IgG-negative), and a cold agglutinin titer of 1:32. Initial imaging identified mild splenomegaly (spleen area: 55 cm²), and bone marrow biopsy excluded lymphoproliferative or plasma cell disorders. No MYD88 mutation was detected.

First-line treatment with corticosteroids yielded no benefit. She was subsequently treated with rituximab (375 mg/m² weekly for 4 weeks) with only modest improvement in hemoglobin, which persisted for approximately one year. Between 2018 and 2022, she received two prolonged courses of mycophenolate mofetil without response, despite bone marrow findings of T-cell infiltration. A second rituximab cycle in 2022 was ineffective. Due to persistent anemia and transfusion dependence, she received supportive treatment with erythropoietin and episodic red blood cell transfusions.

**Figure 1:**
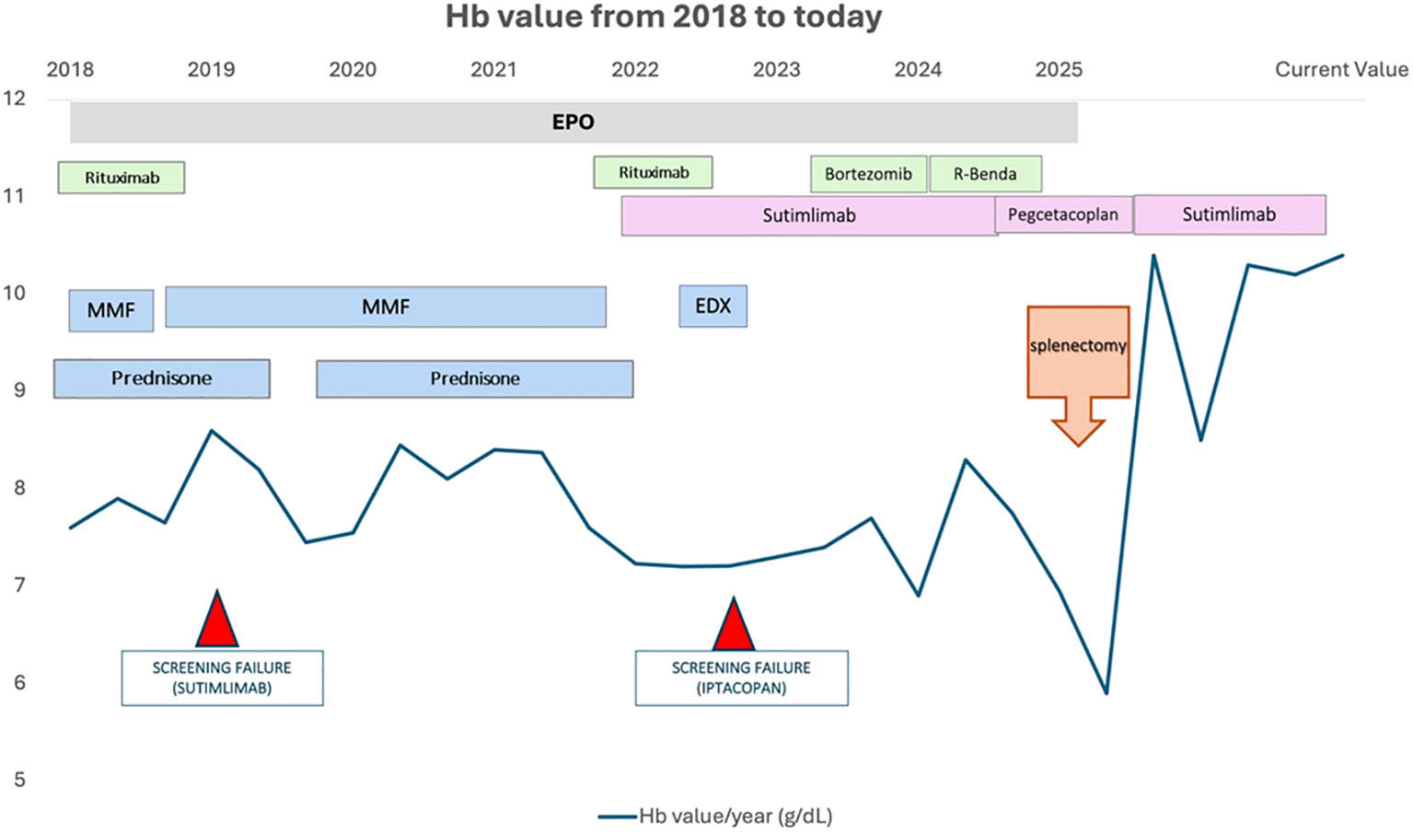
Hemoglobin trend and treatment timeline (2018–2025). Longitudinal hemoglobin levels from 2018 to 2025 plotted alongside all major treatments, including corticosteroids, MMF, rituximab, cyclophosphamide, IVIG, bortezomib, rituximab–bendamustine, sutimlimab, and pegcetacoplan. Screening failures for sutimlimab (2019) and iptacopan (2023) are indicated. A marked and sustained hematologic improvement is observed only after splenectomy in May 2025.

As illustrated in **Figure 1**, hemoglobin levels remained unstable despite multiple therapeutic attempts, with marked improvement only after splenectomy. Also, a simplified representation of the progressive hemoglobin decline, togheter with other hemolytic markers and platelet, over the 2018–2024 period is provided in **Supplementary Figure S1**, confirming a long-term downward trend in hemoglobin levels despite multiple therapeutic attempts.

In September 2022, oral cyclophosphamide was initiated but discontinued shortly thereafter due to gastrointestinal intolerance and lack of efficacy. She began intravenous immunoglobulin (IVIG) therapy during episodes of severe anemia, which provided transient increases in hemoglobin lasting 3–4 weeks. The patient was deemed ineligible for two experimental protocols—sutimlimab in 2019 and iptacopan in 2023—due to low cold agglutinin titers.

In May 2023, compassionate use of sutimlimab was approved and initiated. The patient initially responded with a 2 g/dL increase in hemoglobin; however, within two months, she experienced recurrent hemolytic episodes requiring hospitalization and transfusion. From January 2024, acute hemolysis became more frequent, with new-onset thrombocytopenia and leukopenia. She received intensified IVIG regimens (400 mg/kg/day for 5 days or 800 mg/kg/day for 2 days), with limited benefit. In March 2024, bortezomib was administered (1.3 mg/m² on days 1, 4, 8, 11), followed by four cycles of rituximab plus bendamustine in May 2024. Neither resulted in clinical improvement.

**Figure 2:**
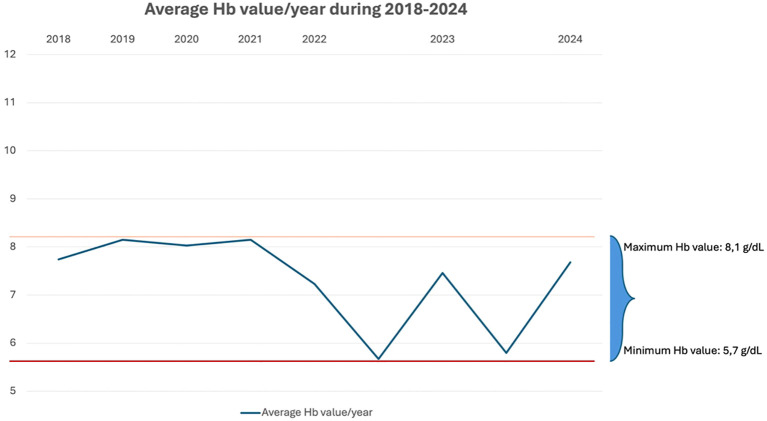
Average yearly hemoglobin values (2018–2024). Annual mean hemoglobin levels showing a progressive decline over time, with relative stability until 2020 followed by a marked decrease from 2021 onward. This trend reflects increasing hemolytic burden and the development of hypersplenism prior to splenectomy.

**Figure 2** shows the progressive decline in average yearly hemoglobin after 2021, consistent with worsening hemolysis and emerging hypersplenism.

Due to worsening pancytopenia, progressive splenomegaly (measuring up to 23 cm in length and 160 cm² in area), and abdominal discomfort, a PET-CT was performed, which ruled out underlying lymphoproliferative disease or transformation. Although imaging findings were negative, definitive histopathological assessment of the spleen following splenectomy further excluded splenic marginal zone lymphoma or other occult B-cell neoplasms, providing additional diagnostic reassurance. In January 2025, sutimlimab was temporarily discontinued due to perceived inefficacy, and pegcetacoplan was introduced biweekly for eight doses. No hematologic improvement was observed, and the patient remained transfusion-dependent. As shown in **Supplementary Figure S2**, bilirubin and LDH progressively increased while reticulocyte and platelet counts declined, suggesting ineffective erythropoiesis combined with hypersplenism.

In March 2025, sutimlimab was resumed. Given persistent symptoms, increasing spleen volume, and hypersplenism-related cytopenias, a multidisciplinary team recommended surgical intervention.

In May 2025, after appropriate preoperative vaccination against encapsulated bacteria, the patient underwent total splenectomy via open laparotomy. The procedure was uneventful, with no intraoperative bleeding complications. The postoperative course was regular, without infectious events, hemorrhagic complications, or thrombotic episodes. The patient was discharged in stable clinical condition. Postoperative labs revealed normalization of leukocyte and platelet counts within one week, and a rapid rise in hemoglobin to 10.4 g/dL. This improvement was so pronounced that clinicians initially questioned the need to continue sutimlimab. However, approximately two weeks after surgery, hemoglobin levels began to decline, suggesting residual hemolytic activity. Continuation of sutimlimab thereafter stabilized hemoglobin and prevented further transfusion requirements, supporting a complementary role of the drug in maintaining remission. Clinical symptoms, including fatigue and cold intolerance, improved significantly.

## 3 Methods

This is a retrospective, single-patient case report based on clinical data collected during routine diagnostic and therapeutic management. The patient provided written informed consent for publication of anonymized clinical information, including laboratory results, imaging, and treatment timeline. All procedures were performed in accordance with institutional standards and the Declaration of Helsinki.

As this is a non-interventional case report involving a single patient, ethical approval from the institutional review board (IRB) was not required, in accordance with local regulatory policies. Diagnostic work-up and therapeutic decisions were guided by current international recommendations for autoimmune hemolytic anemias.

Clinical data were extracted from the patient’s electronic medical record and laboratory information system. A targeted literature review was conducted using PubMed and Scopus databases, limited to the past ten years, with the keywords: “cold agglutinin disease”, “splenectomy”, “complement inhibitors”, “hypersplenism”, and “treatment failure”.

## 4 Discussion

This case illustrates that hypersplenism may significantly contribute to apparent treatment failure in CAD. Despite the sequential administration of all major therapeutic options—including anti-CD20 therapy, immunochemotherapy, and complement inhibition with sutimlimab and pegcetacoplan—the patient experienced persistent cytopenias and progressive splenomegaly. Hematologic recovery was observed only after splenectomy and reintroduction of sutimlimab, suggesting a potential synergistic interaction or, alternatively, that hypersplenism had previously masked pharmacologic efficacy. The temporal dynamics of response in our patient further support this interpretation. The immediate post-splenectomy rise in hemoglobin was so robust that the need for continued sutimlimab was briefly questioned. However, a subsequent decline in hemoglobin within two weeks highlighted the persistence of complement-mediated hemolysis, which was effectively controlled upon resuming sutimlimab.

As shown in **Supplementary Figures S1, S2**, both the long-term trajectory and the acute fluctuations in hemoglobin values support the concept of progressive disease instability leading up to splenectomy. Taken together with **Figure 1** and **2**, these trends reinforce the presence of a dual pathogenic mechanism, in which complement-mediated hemolysis and hypersplenism act synergistically to drive treatment refractoriness. In the present case, the splenic predominance of cytopenias was most consistent with progressive hypersplenism rather than with a change in antibody isotype. The marked increase in spleen size, together with the development of pancytopenia and the rapid normalization of leukocyte and platelet counts following splenectomy, strongly supports a compartmental sequestration mechanism. Although a mild IgG monoclonal component was detected, available clinical and laboratory data did not indicate a shift in the underlying immunopathologic process. Hypersplenism therefore appears to have been the principal driver of splenic sequestration and treatment refractoriness in this patient.

CAD with progressive splenomegaly and cytopenias has already been described, hypothesizing that splenic sequestration may exacerbate cytopenias and suggesting the utility of splenectomy in some cases (4). Okamoto et al. reported a patient with severe anemia and massive splenomegaly who achieved remission following splenectomy alone (7). Similarly, another study described a case of cold agglutinin syndrome secondary to splenic marginal zone lymphoma, in which splenectomy resolved cytopenias despite minimal hemolytic symptoms (8).

Current international guidelines recommend against splenectomy in CAD. The European Hematology Association (9) and the EBMT Handbook (10) both prioritize targeted pharmacologic therapies and reserve splenectomy for exceptional cases with refractory cytopenias or confirmed hypersplenism. Likewise, the use of splenectomy for immune-mediated cytopenias other than CAD has declined in the era of biologics but may retain value in specific clinical contexts (11).

This consideration is especially relevant in older or comorbid patients, such as those with CAD. An 81% complete remission rate has been reported in AIHA patients aged >60 years following splenectomy, although with an increased risk of complications such as infections and thromboses (12).

In summary, although splenectomy is not part of the standard CAD treatment algorithm, it may unmask or enhance the effects of complement inhibition in the rare patients with hypersplenism and complex disease profiles. Individualized evaluation remains essential in these rare and therapeutically challenging cases.

## 5 Conclusions

This case highlights the importance of recognizing hypersplenism as a potential contributor to treatment refractoriness in CAD. Despite exposure to many available therapeutic options—including anti-CD20 therapy, immunochemotherapy, and complement inhibition—the patient experienced persistent cytopenias and progressive splenomegaly, with hematologic recovery achieved only after splenectomy and continuation of sutimlimab. The clinical and laboratory trajectory supports a dual pathogenic mechanism, in which hypersplenism not only aggravated cytopenias but also masked the pharmacologic efficacy of complement inhibition.

While splenectomy is not included in current CAD treatment guidelines due to the hepatic predominance of hemolysis, this case suggests that selected patients with clear evidence of hypersplenism may benefit from surgical intervention, particularly when combined with targeted complement blockade. The observed improvement underscores the need for individualized management strategies in complex or refractory CAD phenotypes. In particular, systematic evaluation of spleen size and cytopenias suggestive of hypersplenism should be considered in CAD patients with treatment failure, as recognition of this condition may significantly influence therapeutic decision-making.

Given the rarity of CAD complicated by hypersplenism, large prospective trials may be difficult to conduct. Collaborative multicenter registries and prospective observational studies may represent more feasible approaches to better define clinical predictors of hypersplenism-driven refractoriness and to clarify the potential complementary role of splenectomy within the evolving therapeutic landscape of CAD.

## Data Availability

The original contributions presented in the study are included in the article/**Supplementary Material**. Further inquiries can be directed to the corresponding author.
