# Supplementary material for: Case Report: Refractory cold agglutinin disease with hypersplenism: efficacy of splenectomy in a patient treated with sutimlimab
Source: Front Immunol. 2026 Mar 30;17:1770676. doi: 10.3389/fimmu.2026.1770676 (PMC13071000; doi:10.3389/fimmu.2026.1770676)
Supplement: Supplementary file 1 [file Supplementaryfile1.docx]

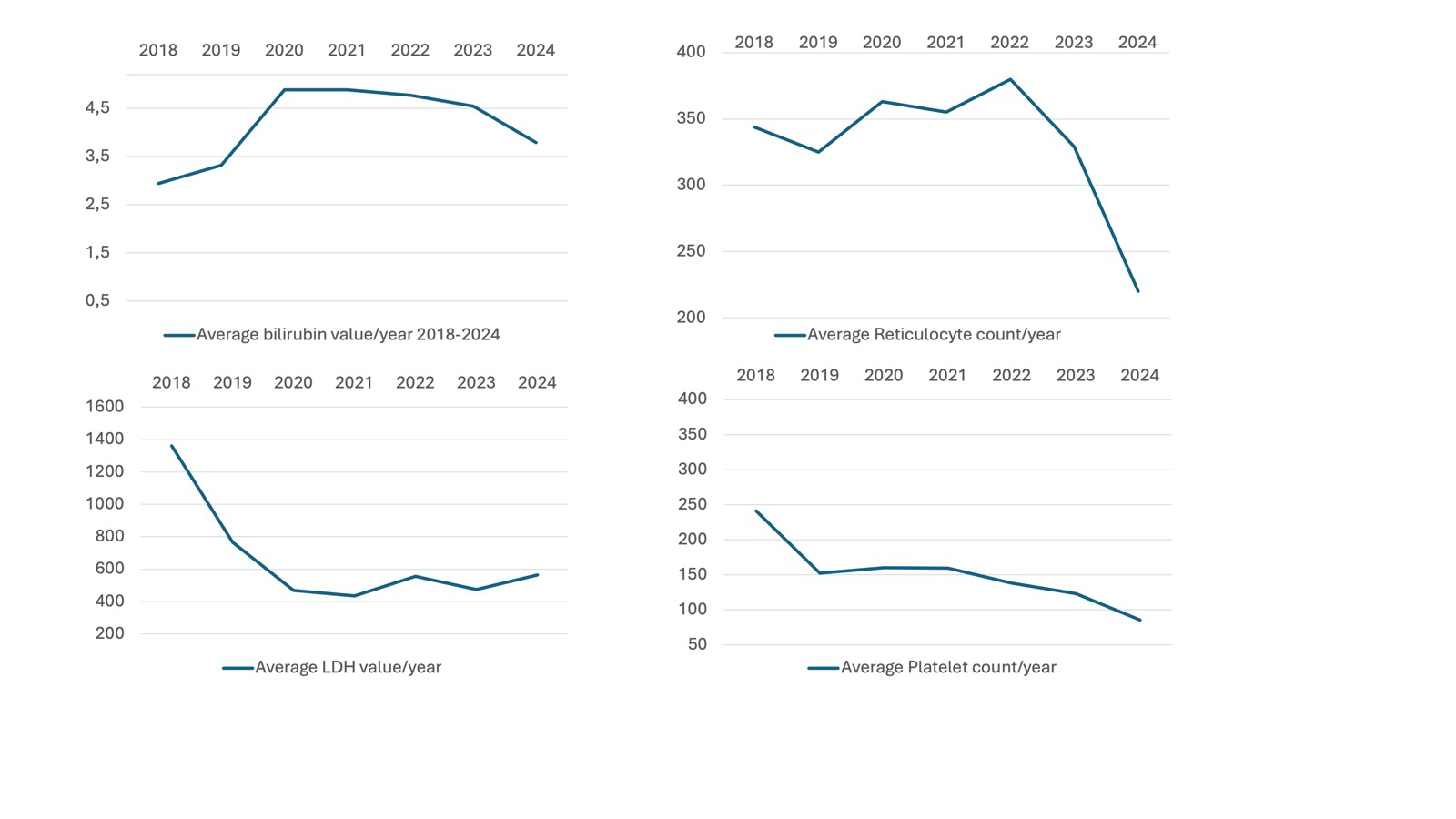
**Supplementary Figure S1.** Simplified annual hemolytic markers and platelet trend (2018–2024).
Linear representation of average yearly without treatment annotations.


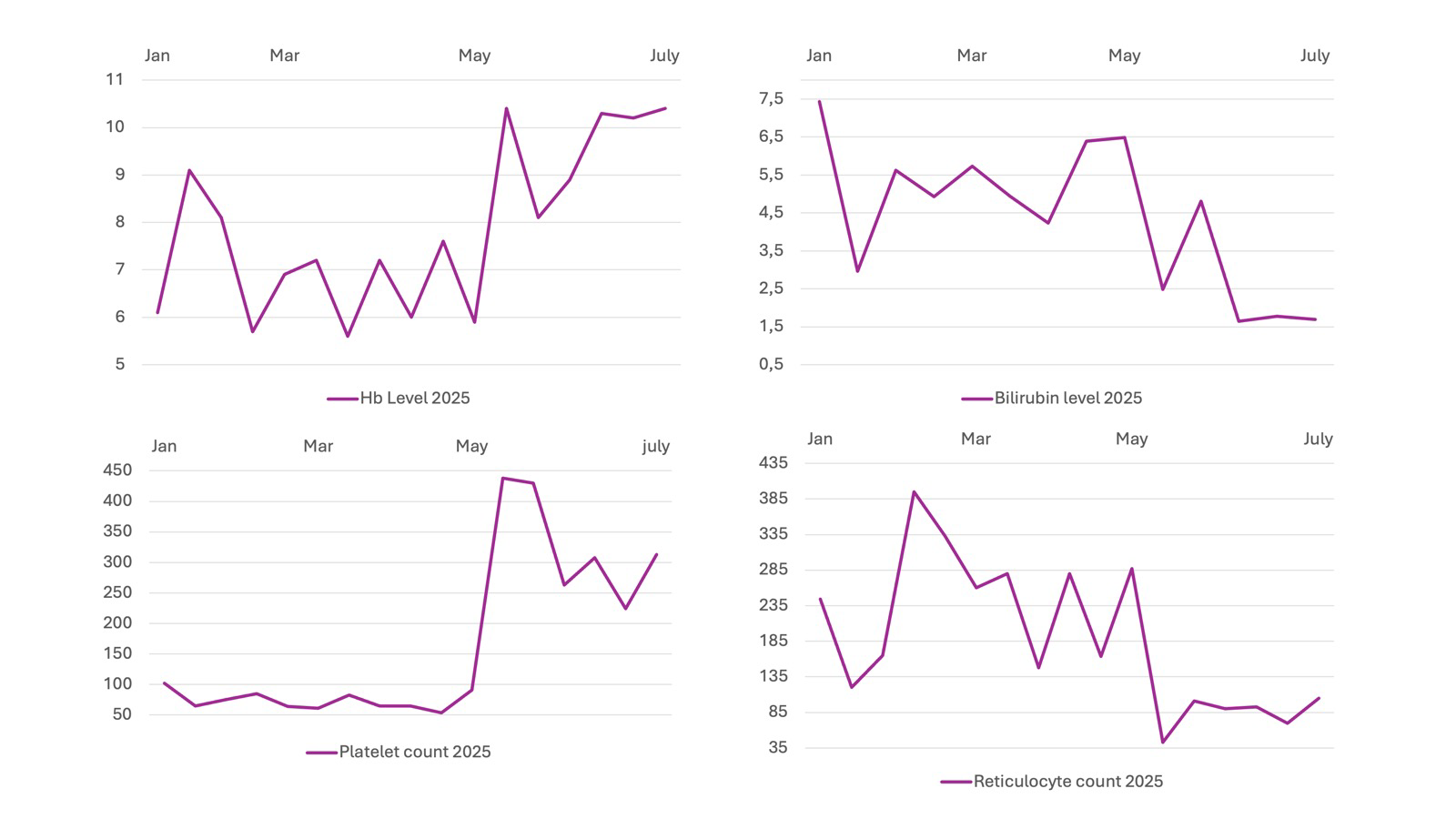


**Supplementary Figure S2**. Weekly hemoglobin and other hemolytic markers and platelet fluctuations during 2025. Detailed weekly values showing marked instability and improvement after splenectomy.
